# Disentangling Phylogenetic Relationships in a Hotspot of Diversity: The Butterworts (*Pinguicula* L., Lentibulariaceae) Endemic to Italy

**DOI:** 10.1371/journal.pone.0167610

**Published:** 2016-12-28

**Authors:** Olga De Castro, Michele Innangi, Antonietta Di Maio, Bruno Menale, Gianluigi Bacchetta, Mathias Pires, Virgile Noble, Giovanni Gestri, Fabio Conti, Lorenzo Peruzzi

**Affiliations:** 1 Dipartimento di Biologia, Università degli Studi di Napoli Federico II, Napoli, Italy; 2 Dipartimento di Scienze e Tecnologie Ambientali, Biologiche e Farmaceutiche, Seconda Università degli Studi di Napoli, Caserta, Italy; 3 Centro Conservazione Biodiversità, Dipartimento di Scienze della Vita e dell’Ambiente, Università degli Studi di Cagliari, Cagliari, Italy; 4 Conservatoire Botanique National Méditerranéen, Hyères, France; 5 Abitazione, Prato, Italy; 6 Scuola di Bioscienze e Medicina Veterinaria, Università di Camerino—Centro Ricerche Floristiche dell’Appennino, Parco Nazionale del Gran Sasso e Monti della Laga, Barisciano (L’Aquila), Italy; 7 Dipartimento di Biologia, Università degli Studi di Pisa, Pisa, Italy; Saint Mary's University, CANADA

## Abstract

The genus *Pinguicula* (Lentibulariaceae) consists of about 100 carnivorous species, also known as butterworts. Eleven taxa are endemic to Italy, which represents a biodiversity hotspot for butterworts in Europe. The aim of our study was to provide a phylogenetic framework for the Italian endemics, in order to: a) investigate the relationships between species in this group; b) evaluate their actual taxonomic value. To achieve this, we analysed all the taxa endemic to Italy, along with several other species, by means of ITS nrDNA analysis. Our results clarify the relationships between Italian endemics and other *Pinguicula* taxa identifying a basal polytomy defined by five clades. All of the Italian endemics (with the exception of *P*. *lavalvae*) fall within a single large clade, which includes *P*. *vulgaris* and allied species. Among them, *P*. *poldinii* represents the most isolated lineage. Other taxa show strong molecular similarities and form a single subclade, although their taxonomic ranks can be retained. *Pinguicula lattanziae* sp. nov., seemingly endemic to Liguria (NW Italy), is also described.

## Introduction

The genus *Pinguicula* L. (butterworts; Lentibulariaceae—Lamiales) includes about 100 species [1]. Butterworts are carnivorous plants that capture their prey by means of mucilaginous and sticky leaves, a rather simple trap compared to the other genera within the family, i.e. *Genlisea* A.St.-Hil. and *Utricularia* L. [2]. The geographic range of *Pinguicula* has two main areas of diversity, one in the Holarctic and the other in the Neotropic floristic kingdoms [3]. Only a few species cover a large geographic range (e.g. *P*. *vulgaris* L. or *P*. *alpina* L.), while many others are endemic to more restricted areas (e.g. *P*. *balcanica* Casper in the Balkans) or narrow endemics (e.g. *P*. *sehuensis* Bacch., Cannas & Peruzzi in a single mountain of Sardinia). Most of the European species grow in moist rocky habitat, with a few exceptions thriving in bogs or damp meadows (e.g. *P*. *corsica* Bern. & Gren. ex Gren. & Godr. or *P*. *lusitanica* L.), however all species require a humid environment [4].

Traditionally, butterworts have been grouped according to features of their generative and vegetative rosettes. *Pinguicula* species show either a temperate growth type, forming *hibernacula* to overcome the cold season, or a tropical growth type, with an overwintering vegetative rosette [3]. Moreover, the generative and vegetative rosettes can be different in shape and/or size, allowing a distinction between homoblastic (homophyllous) and heteroblastic (heterophyllous) species [5]. Another important diagnostic feature in butterworts is their chromosome number, and five different basic chromosome numbers, *x* = 6, 8, 9, 11, 14, have been identified [6]. Casper [7] divided the genus into three subgenera and 15 sections, but his taxonomy resulted artificial in several cases [8]. Compared with other families, a good molecular phylogenetic knowledge is available in literature for Lentibulariaceae [2, 9, 10, 11] and for its three genera: *Pinguicula*, *Genlisea* [12] and *Utricularia* [13].

**Fig 1:**
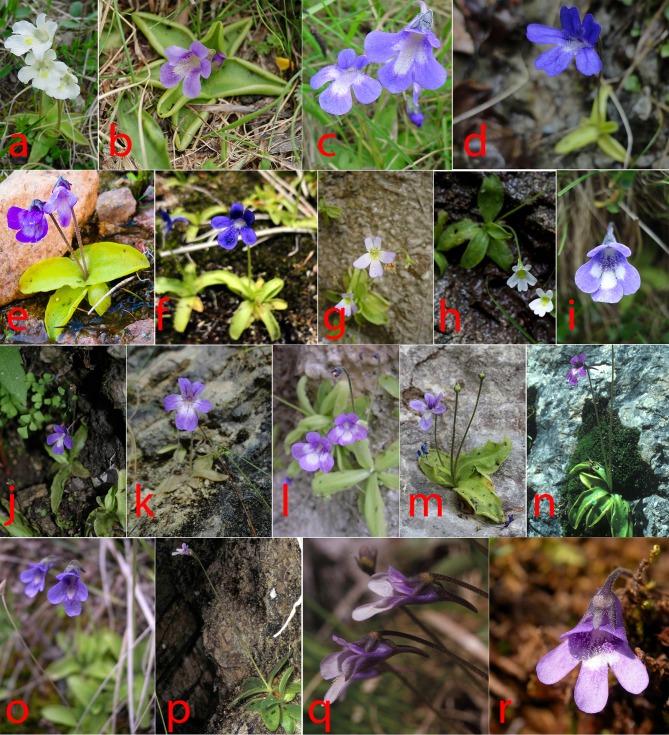
Flower diversity of *Pinguicula* species occurring in Italy and surrounding areas. a = *P*. *alpina*; b = *P*. *apuana*; c = *P*. *balcanica*; d = *P*. *christinae*; e = *P*. *corsica*; f = *P*. *fiorii*; g = *P*. *hirtiflora*; h = *P*. *lavalvae*; k = *P*. *poldinii*; i = *P*. *leptoceras*; j = *P*. *mariae*; l = *P*. *reichenbachiana*; m = *P*. *sehuensis*; n = *P*. *vallis-regiae*; o = *P*. *vulgaris* subsp. *anzalonei*; p = *P*. *vulgaris* subsp. *ernica*; q = *P*. *vulgaris* subsp. *vestina*; r = *P*. *vulgaris* subsp. *vulgaris*.

Phylogenetic reconstructions in *Pinguicula* have been published by Cieslak *et al*. [8], by means of plastid *mat*K and *trn*K group II intron (cpDNA), and by Degtjareva *et al*. [14] by means of ITS region (nuclear internal transcribed spacers and small ribosomal gene, ITS_T_, nrDNA). The ITS_T_ resulted much more informative than plastid markers, and was also used in a couple of studies focused on the species from Northern hemisphere [15] and focused on central American and Cuban taxa [16, 17]. In the above-cited studies, a few Italian endemic species, such as *P*. *fiorii* Tammaro & Pace (endemic to Majella, Abruzzo [18]), *P*. *poldinii* J.Steiger & Casper (endemic to NE Italy [19]), were already included. However, after these studies, many new taxa were described from Italy, such as *P*. *vallis-regiae* F.Conti & Peruzzi endemic to Camosciara (Abruzzo, central Italy), *P*. *vulgaris* L. subsp. *anzalonei* Peruzzi & F.Conti endemic to central Italy (Latium), *P*. *vulgaris* subsp. *ernica* Peruzzi & F.Conti endemic to Ernici Mountains (Abruzzo, central Italy), *P*. *vulgaris* subsp. *vestina* F.Conti & Peruzzi endemic to Gran Sasso (Abruzzo, central Italy) [20], *P*. *apuana* Ansaldi & Casper and *P*. *mariae* Casper endemic to Apuan Alps (Tuscany, central Italy) [21], *P*. *christinae* Peruzzi & Gestri endemic to N Apennine (northern Italy) [22], *P*. *lavalvae* Innangi & Izzo [23] endemic to Mts. Picentini (Campania, southern Italy), and *P*. *sehuensis* Bacch., Cannas & Peruzzi endemic to Sardinia [1]. With a total of 11 endemic taxa [24, 25], Italy clearly represents a biodiversity hotspot for this genus in Europe (Fig 1). To date, the biogeographic and evolutionary history of butterworts is still not completely resolved [3, 14]. Hence, the aim of our study was to provide a phylogenetic framework for the Italian endemics, in order to: a) investigate the relationships between species in this group; b) evaluate their actual taxonomic value. To achieve this, we analysed all butterworts endemic to Italy, along with several other species by means of nuclear molecular markers and literature data. The molecular marker chosen for this study was the ITS_T_ (nrDNA), which proved to be the best tool for our purposes. Our marker choice is supported by: (1) high variability and discriminating capabilities of this molecular marker among *Pinguicula* taxa as already shown by Degtjareva *et al*. [14], Kondo & Shimai [15], Shimai & Kondo [16] and Shimai *et al*. [17]; (2) a complete GenBank nrDNA-*Pinguicula* database to be used for an exhaustive phylogenetic reconstruction; (3) completeness of information as a consequence of the biparental inheritance of nrDNA [6, 26, 27, 28]. In fact, cpDNA markers may cause confusion in inferring phylogenetic relationships in potentially hybrid/introgressed/polypoid taxa (e.g. [29]) because, with few exceptions (e.g. [30, 31]) they are maternally inherited [32, 33, 34] and hybridization phenomena easily go undetected. In addition, while cpDNA markers were successful in resolving phylogenetic relationships among Central American species [8], their variability resulted very low within *P*. sect. *Pinguicula* (including most of the species studied here), possibly due to more recent speciation events.

## Materials and Methods

### Nomenclature

The electronic version of this article in Portable Document Format (PDF) in a work with an ISSN or ISBN will represent a published work according to the International Code of Nomenclature for algae, fungi, and plants, and hence the new names contained in the electronic publication of a PLoS ONE article are effectively published under that Code from the electronic edition alone, so there is no longer any need to provide printed copies.

In addition, new names contained in this work have been submitted to IPNI, from where they will be made available to the Global Names Index. The IPNI LSIDs can be resolved and the associated information viewed through any standard web browser by appending the LSID contained in this publication to the prefix http://ipni.org/. The online version of this work is archived and available from the following digital repositories: PubMed Central, LOCKSS, ResearchGate (https://www.researchgate.net/), ARPI (https://arpi.unipi.it/).

### Ethics statement

The plant tissues (a small portion of leaf) analyzed are from herbarium specimens. These specimens are stored in Herbaria. In addition, when the plants were sampled, no specific permissions were required for this. Finally, the sampling has been performed without damaging the populations or the species in the case of narrow endemics with a single known population.

### *Pinguicula* sampling

The plant tissues (i.e. small portion of leaf) analyzed were sampled from herbarium specimens. These source of material and Herbaria is summarized in Table 1.

**Table 1:** List of *Pinguicula* accessions sequenced in the molecular study, available karyological information, voucher information, origin, ITS_T_—GC content (%) and GenBank accession no.

Code	Taxon	2*n* (ref.)	Origin (lat./long.) (date)	Voucher (Herbarium) (DNA code)	ITS_T_	
					GC%	GenBank no.
P1	*Pinguicula apuana* Casper & Ansaldi	64, 128 [35]	Italy, Tuscany, Apuan Alps, M. Corchia (44.03, 10.29) (24 June 2004)	*M*. *Ansaldi*, *G*. *Bedini*, *G*. *Cataldi* (PI) (Ei 2)	65.6	LN887909
P2	*P*. *apuana*	64, 128 [35]	Italy, Tuscany, Apuan Alps, M. Corchia (44.03, 10.29) (24 June 2004)	*M*. *Ansaldi*, *G*. *Bedini*, *G*. *Cataldi* (PI) (Ei 3)	65.6	LN887910
P3	*P*. cf. *apuana*	64 [36]	France, Maritime Alps, Bendola (43.97, 7.58) (19 June 2013)	*M*. *Pires* (CBNMed) (Ei 23)	65.6	LN887911
P4	*P*. cf. *apuana*	64 [36]	France, Maritime Alps, Bendola (43.97, 7.58) (19 June 2013)	*M*. *Pires* (CBNMed) (Ei 24)	65.6	LN887912
P5	*P*. *balcanica* Casper	32 [7]	Bulgaria, M. Pirin, 2000 m (41.61, 23.54) (26 June 2006)	*L*. *Peruzzi*, *D*. *Uzunov*, *G*. *Caruso* (PI) (Ei 4)	66	LN887913
P6	*P*. *balcanica*	n.a.	Bulgaria, M. Vitosha, 1700–1800 m (42.59, 23.27) (23 June 2006)	*L*. *Peruzzi* (PI) (Ei 5)	66	LN887914
P7	*P*. *christinae* Peruzzi & Gestri	n.a.	Italy, Tuscany, N Apennine, Foce di Campolino, 1600 m (44.10, 10.64) (4 July 2011)	*L*. *Peruzzi*, *G*. *Gestri* (PI) (Ei 6)	65.6	LN887915
P8	*P*. *christinae*	64 [7]	Italy, Tuscany, N Apennine, Lama Rossa, 1460 m (44.21, 10.38) (2 June 2011)	*G*. *Gestri* (PI) (Ei 7)	65.4	LN887916
P9	*P*. *christinae*	n.a.	Italy, Tuscany, N. Apennine, Val di Luce, 1620 m (44.12, 10.63) (13 June 2010)	*G*. *Gestri* (PI) (Ei 9)	65.6	LN887917
P10	*P*. cf. *christinae*	n.a.	Italy, Liguria, N. Apennine, Val d’Aveto, M. Aiona (44.47, 9.45) (21 June 2014)	*G*. *Gestri* (PI) (Ei 35)	65.6	LN887918
P11	*P*. *corsica* Bernard & Gren. ex Gren. & Godr.	n.a.	France, Corsica, Val d’Asco (42.42, 8.96) (25 September 2013)	*G*. *Bacchetta* 2051318 (CAG) (Ei 31)	63.9	LN887919
P12	*P*. *corsica*	n.a.	France, Corsica, Val d’Asco (42.42, 8.96) (25 September 2013)	*G*. *Bacchetta* 2051303 (CAG) (Ei 32)	63.9	LN887920
P13	*P*. *corsica*	n.a.	France, Corsica, Val d’Asco (42.42, 8.96) (25 September 2013)	*G*. *Bacchetta* 2051312 (CAG) (Ei 33)	63.9	LN887921
P14	*P*. *corsica*	n.a.	France, Corsica, Val d’Asco (42.42, 8.96) (25 September 2013)	*G*. *Bacch*etta 2051316 (CAG) (Ei 34)	63.9	LN887922
P15	*P*. *fiorii* Tammaro & Pace	n.a.	Italy, Abruzzo, Majella, Fara S. Martino (42.08, 14.18) (23 June 1988)	*F*. *Conti 12720* (APP) (Ei 15)	65.6	LN887923
P16	*P*. *fiorii*	n.a.	Italy, Abruzzo, Majella, Valle dell’Orfento (42.17, 14.08) (28 May 2013)	*L*. *Peruzzi* (PI) (Ei 25)	65.6	LN887924
P17	*P*. *fiorii*	n.a.	Italy, Abruzzo, Majella, Valle dell’Orfento (42.17, 14.08) (28 May 2013)	*L*. *Peruzzi* (PI) (Ei 26)	65.6	LN887925
P18	*P*. *hirtiflora* Ten.	n.a.	Italy, Campania, Mts. Picentini, Matrunolo gorge (40.83, 14.92) (June 2010)	*M*. *Innangi*, *A*. *Izzo VM217* (NAP) (Ei 38)	56	LN887926
P19	*P*. *hirtiflora*	n.a.	Italy, Campania, Amalfi peninsula, Vecite gorge (40.67, 14.66) (8 July 2010)	*M*. *Innangi*, *A*. *Izzo VV14* (NAP) (Ei 39)	56	LN887927
P20	*P*. *hirtiflora*	28 [7]	Italy, Campania, Sorrento peninsula, Mt. S’Angelo Tre Pizzi (40.65, 14.50) (17 June 2010)	*M*. *Innangi*, *A*. *Izzo MSA523* (NAP) (Ei 36)	56	LN887928
P21	*P*. *hirtiflora*	28 [7]	Italy, Calabria, Rossano Calabro (39.57, 16.64) (2011)	(CLU) (Ei 40)	55.5	LN887929
P22	*P*. *hirtiflora*	28 [7]	Italy, Calabria, Rossano Calabro (39.57, 16.64) (16 June 2012)	*M*. *Innangi*, *A*. *Izzo*, *P*. *Sbragia n*.*s*. (NAP)	55.5	LN887930
P23	*P*. *hirtiflora*	n.a.	Greece, N Pindo, along the road from Grevena to Metsovo, 1000 m (39.79, 21.27) (27 June 2006)	*L*. *Peruzzi*, *D*. *Uzunov*, *G*. *Caruso* (PI) (Ei 11)	55.4	LN887931
P24	*P*. *hirtiflora*	28 [67]	France, Maritime Alps, Val Roya, gorges de Bergue, along the road from Fontan to St. Dalmas de Tende, 500 m (44.01, 7.56) (30 April 2007)	*L*. *Peruzzi*, *K*.*F*. *Caparelli* (PI) (Ei 12)	55.4	LN887932
P25	*P*. *lavalvae* Innangi & Izzo	n.a	Italy, Campania, Mts. Picentini, Sabato valley (40.79, 14.97) (June 2010)	*M*. *Innangi*, *A*. *Izzo VS15* (NAP)	56	LN887933
P26	*P*. *lavalvae*	n.a.	Italy, Campania, Mts. Picentini, Sabato valley (40.79, 14.97) (June 2010)	*M*. *Innangi*, *A*. *Izzo VS21* (NAP) (Ei 37)	56	LN887934
P27	*P*. *mariae* Casper	32 [7]	Italy, Tuscany, Apuan Alps, Isola Santa (44.06, 10.31) (27 April 2004)	*M*. *Ansaldi* (PI) (Ei 13)	65.7	LN887935
P28	*P*. *poldinii* J.Steiger & Casper	32 [7]	Italy, Friuli Venezia Giulia, San Francesco (Pordenone), on the left side of Arzino (46.33, 12.93) (29 April 2013)	*L*. *Peruzzi*, *G*. *Gestri*, *B*. *Pierini* (PI) (Ei 27)	63.4	LN887936
P29	*P*. *poldinii*	32 [7]	Italy, Friuli-Venezia Giulia, San Francesco (Pordenone), on the right side of Arzino (46.30, 12.93) (29 April 2013)	*L*. *Peruzzi*, *G*. *Gestri*, *B*. *Pierini* (PI) (Ei 28)	63.4	LN887937
P30	*P*. *reichenbachiana* Schindl.	32 [37]	France, Maritime Alps, Val Roya, Gorges de Bergue, along the road from Fontan to St. Dalmas de Tende, 500 m (44.01, 7.56) (30 April 2007)	*L*. *Peruzzi*, *K*.*F*. *Caparelli* (PI) (Ei 14)	65.6	LN887938
P31	*P*. *seuhensis* Bacch., Cannas & Peruzzi	16 [1]	Italy, Sardinia, Cengia del Monte Tonneri, Seui (39.89, 9.38) (3 May 2014)	*G*. *Bacchetta 471313* (CAG) (Ei 29)	63.8	LN887939
P32	*P*. *seuhensis*	16 [1]	Italy, Sardinia, Cengia del Monte Tonneri, Seui (39.89, 9.38) (3 May 2014)	*G*. *Bacchetta 471314* (CAG) (Ei 30)	63.8	LN887940
P33	*P*. *vallis-regiae* F.Conti & Peruzzi	n.a.	Italy, Abruzzo, Villetta Barrea (41.78, 13.90) (2 July 2004)	*F*. *Conti 11290* (APP) (Ei 16)	65.5	LN887941
P34	*P*. *vulgaris* L. subsp. *anzalonei* Peruzzi & F.Conti	n.a.	Italy, Lazio, Mt. Simbruini, Piscicarello di Jenne (41.90, 13.14) (26 May 2005)	*F*. *Conti*, *F*. *Bartolucci*, *A*. *Bernardini 21422* (APP) (Ei 17)	65.8	LN887942
P35	*P*. *vulgaris* subsp. *ernica* Peruzzi & F.Conti	64 [38]	Italy, Abruzzo, Mt. Ernici, Zompo Lo Schioppo (41.84, 13.39) (4 July 2004)	*F*. *Conti*, *F*. *Bartolucci*, *M*. *Iocchi 21421* (APP) (Ei 18)	65.8	LN887943
P36	*P*. *vulgaris* subsp. *vestina* F. Conti & Peruzzi	64 [38]	Italy, Abruzzo, Gran Sasso, Valle del Rio Arno (42.49, 13.54) (6 July 2005)	*F*. *Conti*, *F*. *Bartolucci 10920* (APP) (Ei 19)	65.8	LN887944
P37	*P*. *vulgaris* subsp. *vulgaris*	n.a.	Italy, Abruzzo, Monti della Laga (42.65, 13.39) (23 June 2005)	*F*. *Conti*, *F*. *Bartolucci* 20984 (APP) (Ei 21)	65.9	LN887945
P38	*P*. *vulgaris* subsp. *vulgaris*	n.a.	Italy, Abruzzo, Monti della Laga, Pizzo di Moscio (42.65, 13.39) (13 August 2002)	*F*. *Conti 2177* (APP) (Ei 22)	65.9	LN887946

A total of 38 accessions, corresponding to 13 species, was sampled. A single accession from Italy (*P*. cf. *christinae*) exhibited peculiar morphological features and its taxonomic position has been investigated in the present study (see morphometric analyses in S1 File).

Most of the sampled taxa are endemic to Italy, except for those accessions coming from Corsica (*P*. *corsica*), France (*P*. cf. *apuana* from Bendola, *P*. *hirtiflora* from Val Roya, *P*. *reichenbachiana* Schindl. also from Val Roya), Bulgaria (*P*. *balcanica* Casper) and Greece (*P*. *hirtiflora*). Sequence data of further 26 *Pinguicula* species (73 total accessions), many of them with a range not covering Italy, were obtained from GenBank, based on the works of Degtjareva *et al*. [14] and Kondo & Shimai [15] (Table 2).

**Table 2:** List of GenBank ITS_T_ accessions used for the phylogenetic analysis of *Pinguicula* taxa (taxon, distribution, GenBank no., reference).

Taxon	Origin	GenBank no.	Reference
**Outgroup**			
*Antirrhinum majus* L. subsp. *cirrhigerum* (Ficalho) Franco	Morocco, Doukkala-Abda, El Jadida	EU677200	[39]
*A*. *majus* subsp. *linkianum* (Boiss. & Reut.) Rothm.	Spain, La Coruña, Cedeira	EU677214	[39]
*A*. *majus* subsp. *litigiosum* (Pau) Rothm.	Spain, Teruel, Griegos	EU677216	[39]
*A*. *majus* subsp. *majus*	Spain, Gerona, La Molina	EU677219	[39]
*A*. *majus* subsp. *tortuosum* (Vent.) Rouy	Morocco, West Rif, Talembot	EU677242	[39]
*Kigelia africana* (Lam.) Benth.	USA, County Arboretum & Botanic Garden of Los Angels	AY178638	[40]
**Sistergroup to *Pinguicula* L.**			
*Genlisea violacea* St.-Hil.	Brazil, Itacambira, Mato Grosso	AB212116	[15]
*Utricularia intermedia* Hayne	Russia, Tver’ Prov.	DQ225109	[14]
*U*. *minor* L.	Japan, Higashi-Hiroshima, Hiroshima	AB212118	[15]
***Pinguicula* accessions**			
*P*. *agnata* Casper	Germany, Botanical Garden of Jena	DQ441602	[14]
*P*. *alpina* L.	Slovakia, Mala Fatra, Terchová,	AB198341	[15]
*P*. *alpina* (A)	Romania, Transylvanian Alps, W Sinaia	DQ222969	[14]
*P*. *alpina* (B)	Italy, Trentino Alto-Adige, Siusi Alps	DQ438092	[14]
*P*. *alpina* (C)	Switzerland, Canton Bern, between Habkern and Grünenberg-Pass	DQ438100	[14]
*P*. *balcanica* Casper	Bulgaria, S of Sofia	DQ222954	[14]
*P*. *balcanica*	Greece, Vardoússia, Fokída	AB198342	[15]
*P*. *bohemica* Krajina	Czech Republic, SE of Ĉeska Lípa	DQ441597	[14]
*P*. *bohemica*	Czech Republic, Ĉeska Lípa	AB198343	[15]
*P*. *caerulea* Walter	USA, Georgia, SW of Folkston	DQ222963	[14]
*P*. *corsica* Bernard & Gren. ex Gren. & Godr.	France, Corsica, Gorges de la Restonica	AB198344	[15]
*P*. *corsica* (A)	France, Corsica, Lac de Melo	DQ222955	[14]
*P*. *corsica* (B)	France, Corsica, Lac de Melo	DQ438098	[14]
*P*. *corsica* (C)	France, Corsica, Lac de Melo	DQ438090	[14]
*P*. *crystallina* Sm.	Cyprus, Tróodos	AB198363	[15]
*P*. *crystallina* (A)	Cyprus	DQ222965	[14]
*P*. *crystallina* (B)	Cyprus, Kakopetria, Ayios Nicolaos	DQ438082	[14]
*P*. *dertosensis* (Cañig.) Mateo & M.B.Crespo	Spain, Tarragona, Sierra de Caro/Sierra de Fortalesa	DQ441598	[14]
*P*. *dertosensis*	Spain, Ports de Beceit, Terragona	AB198345	[15]
*P*. *fiorii* Tammaro & L.Pace	Italy, Abruzzo, Majella, Bocca di Valle	DQ222952	[14]
*P*. *fiorii*	Italy, Abruzzo, Valle dell’Orfento	AB198346	[15]
*P*. *grandiflora* Lam. subsp. *grandiflora*	France, Pyrenees, Lac de Fabregés,	AB198347	[15]
*P*. *grandiflora* subsp. *grandiflora* f. *grandiflora* (A)	France, Pyrenees, Dept. Hautes	DQ222958	[14]
*P*. *grandiflora* subsp. *grandiflora* f. *grandiflora* (B)	France, Dept. Ain	DQ438099	[14]
*P*. *grandiflora* subsp. *grandiflora* f. *grandiflora* (C)	Spain, Picos de Europa, Rio Care	DQ438091	[14]
*P*. *grandiflora* subsp. *grandiflora* f. *pallida* (Gaudin) Casper (A)	France, Dept. Ain	DQ222957	[14]
*P*. *grandiflora* subsp. *grandiflora* f. *pallida* (B)	France, Dept. Ain	DQ438097	[14]
*P*. *grandiflora* subsp. *rosea* (Mutel) Casper	France, Goncelin, Isère	AB198348	[15]
*P*. *grandiflora* subsp. *rosea* (A)	France, Dept. Isère, between Concelin and Sollières NE of Grenoble	DQ222956	[14]
*P*. *grandiflora* subsp. *rosea* (B)	France, Dept. Isère, Col du Granier	DQ438081	[14]
*P*. *hirtiflora* Ten.	Italy, Campania, Vietri sul Mare	AB198364	[15]
*P*. *hirtiflora* (A)	Italy, Campania, Salerno	DQ222966	[14]
*P*. *hirtiflora* (B)	Greece, Thessalia, Mount Olympus	DQ438083	[14]
*P*. *leptoceras* Rchb.	Italy, Liguria, Col di Tende	DQ222947	[14]
*P*. *leptoceras*	France, Col de Tende	AB198349	[15]
*P*. *longifolia* Ramond ex DC. subsp. *caussensis* Casper	France, Gorges du Tarn	AB198350	[15]
*P*. *longifolia* subsp. *caussensis* (A)	France, Gorge du Tarn	DQ222948	[14]
*P*. *longifolia* subsp. *caussensis* (B)	France, Gorge du Tarn	DQ438095	[14]
*P*. *longifolia* subsp. *caussensis* (C)	France, Gorge du Tarn	DQ438088	[14]
*P*. *longifolia* subsp. *longifolia*	Spain, Huesca, Valle de Ordesa	AB198351	[15]
*P*. *longifolia* subsp. *longifolia* (A)	France, Pyrenees	DQ222959	[14]
*P*. *longifolia* subsp. *longifolia* (B)	France, Central Pyrenees	DQ438089	[14]
*P*. *longifolia* subsp. *reichenbachiana* (Schindler) Casper	France, Maritime Alps, Roya Valley	AB198352	[15]
*P*. *longifolia* subsp. *reichenbachiana* (A)	France, Maritime Alps, Roya Valley	DQ222950	[14]
*P*. *longifolia* subsp. *reichenbachiana* (B)	France, Maritime Alps, Roya Valley	DQ438094	[14]
*P*. *longifolia* subsp. *reichenbachiana* (C)	France, Maritime Alps, Roya Valley	DQ438087	[14]
*P*. *lusitanica* L.	Spain, Rio de la Miel near Algeciras	DQ222960	[14]
*P*. *lusitanica*	England, Brokenhurst, Hampshire	AB198365	[15]
*P*. *lutea* Walter	USA, Alabama, S. Elsauer	DQ222962	[14]
*P*. *macroceras* Link	Japan, Nagano, Todai-gawa	AB198353	[15]
*P*. *macroceras* subsp. *nortensis* J.Steiger & Rondeau	USA, northernmost California, del Norte County	DQ222951	[14]
*P*. *moranensis* Kunth	Germany, Botanical Garden of Jena	DQ222967	[14]
*P*. *mundi* Blanca Jamilena Ruíz Rejón & Reg.Zamora	Spain, Albacete, near border to Prov. Jaen	DQ441599	[14]
*P*. *mundi*	Spain, Albacete, Nacimiento del Río Mundo	AB198354	[15]
*P*. *nevadensis* (H.Lindb.) Casper	Sapin, Sierra Nevada	AB198355	[15]
*P*. *planifolia* Chapm.	USA, Florida, Apalachicola Forest near Sumatra	DQ441601	[14]
*P*. *poldinii* J.Steiger & Casper	Italy, Friuli Venezia Giulia, Campone	DQ441600	[14]
*P*. *poldinii*	Italy, Friuli Venezia Giulia, Val d’Arzino	AB198356	[15]
*P*. *primuliflora* C.E.Wood & R.K.Godfrey	USA, Florida, S Crestvien	DQ222964	[14]
*P*. *ramosa* Miyoshi	Japan, Tochigi, Koshin-zan	AB198357	[15]
*P*. *vallisneriifolia* Webb	Spain, Sierra de Segura	AB198358	[15]
*P*. *vallisneriifolia* (A)	Spain, Sierra de Cazorla, Cueva de la Magdalena	DQ222953	[14]
*P*. *vallisneriifolia* (B)	Spain, Sierra de Cazorla, Cueva de la Magdalena	DQ438084	[14]
*P*. *variegata* Turcz.	Russia, Khabarovskiy kray, near Okhotsk	DQ222968	[14]
*P*. *variegata*	Russia, Sakhalin Island	AB198359	[15]
*P*. *villosa* L.	USA, Alaska, Broad Pass, Kantwell	AB198360	[15]
*P*. *villosa* (A)	Cultivated material (no origin)	DQ222961	[14]
*P*. *villosa* (B)	Norway, Soer-Troendelag	DQ438096	[14]
*P*. *villosa* (C)	Sweden, Abisko	DQ438085	[14]
*P*. *vulgaris* L.	Slovakia, Velká Fatra, Martín	AB198361	[15]
*P*. *vulgaris* (A)	Iceland	DQ222949	[14]
*P*. *vulgaris* (B)	Germany, Altenberga near Jena	DQ438086	[14]
*P*. *vulgaris* (C)	Switzerland, Canton Bern	DQ438093	[14]

### DNA extraction, PCR amplification and sequence analyses

Total DNA was extracted from approx. 3 mg of fresh leaf or 2 mg of dried leaf material using ZR Plant/Seed DNA MicroPrep (ZYMORESEARCH), according to the manufacturer’s protocol, then resuspended in 20 μL sterile distilled water. The concentration was estimated by quantifying 1 μl of DNA extract using a Qubit dsDNA HS Assay Kit with the Qubit ver. 2 Fluorometer (Invitrogen, Thermo Fisher Scientific Inc., Waltham, MA, USA).

ITS_T_ was amplified by using plant universal primers that were reported in the literature {Forward-18S(3’) 5’-GGA GAA GTC GTA ACA AGG TTT CCG-3′, and Reverse-26S(5′)-internal 5’-TTC GCT CGC CGT TAC TAA GGG-3’} [28] and selective internal primers designed on *Pinguicula* sp. accessions from GenBank (Pingui_18Sfor 5’-AAG GAT CAT TGT CGA DWY Y-3’ and Pingui_26Srev 5’-TGR GGT CGC RRR IGT TGG CR-3’). The use of nested-PCR with selective primers for *Pinguicula* accessions can be very useful to remove possible contaminators (e.g. pollen) occurring on the flypaper traps. All oligos were synthetized by Macrogen Inc., the annealing temperature was 55°C for the two primer pairs. The volume of each polymerase chain reaction (PCR) was 25 μl, with *c*. 10–20 ng of DNA template, 2.5 μl of 10× DreamTaq Buffer (Thermo Fisher Scientific Inc.), 0.5 μl each of the 2.5 mM nucleotides (Promega), 0.125 μl of 50mM primer and 0.25 μl DNA DreamTaq polymerase (5Uμl^–1^) (Thermo Fisher Scientific Inc.). Amplifications of recalcitrant DNA templates were performed by using nested-PCRs with internal selective primers or KAPA3G Plant PCR Kit (KAPABIOSYSTEMS). The cycling parameters of the PCRs were performed according to the manufacturer’s instructions in a SimpliAmp thermal cycler (Applied Biosystems, Thermo Fisher Scientific Inc.). Amplification products were purified, using the NucleoSpin® Gel and PCR Clean-up (Macherey-Nagel) following the manufacturer’s protocols. An aliquot of approximately 70 ng of purified DNA template was used with the Bright Dye Terminator Cycle Sequencing Kit (ICloning) following the procedure according to Di Maio & De Castro [41] and analysed with a 3130 Genetic Analyzer (Applied Biosystems, Thermo Fisher Scientific Inc.).

Several sequences with slippage events for a mononucleotide repeat (polyC/G) were cloned with the CloneJET PCR Cloning Kit (Thermo Fisher Scientific Inc.) following the protocol of the manufacturer. Transformation was carried out using StrataClone SoloPack Competent Cells (Agilent Technologies). Bacteria were cultured in LB medium at 37°C for 30 min and then on LB agar plates containing 100 ug/ml ampicillin. Eight randomly selected clones from each transformation were amplified and sequenced with the pJET2.1 forward and pJET2.1 reverse primers (Thermo Fisher Scientific Inc.) which matching in the flanking regions of the pJET1.2/blunt vector polylinker.

Complete sequences of both strands of each PCR product were processed using the AB DNA Sequencing Analysis ver. 5.2 (Applied Biosystems, Life Technologies), and visually checked using Chromas Lite ver. 2.1.1. software (Chromas Lite version 2.1, http://technelysium.com.au/?page_id=13). Then, sequences were aligned using ClustalW ver. 1.4 software [42] as daughter processes of BioEdit ver. 7.2.5 software [43]. The aligned sequences were visually inspected to correct gap distributions devoid of biological meaning. The sequences obtained in this study are available from GenBank and accession numbers are provided in Table 1.

### Phylogenetic analyses

One hundred and twenty accessions were analysed using two phylogenetic approaches: Bayesian inference (BI) and maximum parsimony (MP). The trees generated by these methods were checked for congruence. Outgroups were chosen after several preliminary analyses considering the literature data [7, 14, 15]: *Kigelia africana* (Lam.) Benth. {GenBank (GB): AY178638}, *Antirrhinum majus* L. (GB: EU677219) and some of its subspecies {i.e. *A*. *majus* subsp. *linkianum* (Boiss. & Reut.) Rothm., GB: EU677214; *A*. *majus* subsp. *cirrhigerum* (Ficalho) Franco, GB: EU677200; *A*. *majus* subsp. *tortuosum* (Vent.) Rouy, GB: EU677242; *A*. *majus* subsp. *litigiosum* (Pau) Rothm., GB: EU677216}. Representative of *Pinguicula* sister group were also included: *Genlisea violacea* St.-Hil. (GB: AB212116), *Utricularia intermedia* Hayne (GB: DQ225109) and *U*. *minor* L. (GB: AB212118).

The BI method for phylogenetic reconstruction was implemented using MrBayes ver. 3.2.2 software [44, 45] and jModelTest ver. 2.1.4 software to obtain the best nucleotide substitutions model [46, 47]. Preliminary analyses were performed to obtain the optimal BI settings. Bayesian Markov Chain Monte Carlo (MCMC) algorithm was run for 15,000,000 generations discarding the first 12% generations (burninfrac = 0.12). Four runs were performed and two heated chains were used. Bayesian posterior probabilities (PP) were obtained from the 50% majority rule consensus of 52,800,000 trees (13,200,000 trees from each runs). The general time reversible (GTR) + proportion of invariant sites (I) + gamma distribution (G) model was used in the analyses (set nst = 6 rates = invgamma), according to the results obtained with jModelTest under the Akaike Information Criterion (AIC) [48]. The same model was obtained using also the correction for sample size (AICc).

MP reconstruction were performed with TNT ver. 1.1 software [49, 50]. Settings included: gaps scored as missing data; maximum storage space of 99,000 trees; a tree storage space per iteration of 100; 100 iterations branch swapping via tree bisection-reconnection. In addition, we used also the "new technology search" (which is guaranteed to find at least one shortest tree), setting 10 hits to the shortest length and an initial level of 100. Bootstrap values [51] were calculated from 1,000 replicates.

## Results

### Molecular analysis

The alignment of ITS_T_ sequences of all *Pinguicula* accessions was relatively straightforward due to not complex variation among sequences (598–625 bp, *P*. *caerulea* Walter-*P*. *agnata* Casper/*P*. *moranensis* Kunth). In the 38 accessions of *Pinguicula* sequenced in the present study, the length of the ITS_T_ ranged from 607 (*P*. *hirtiflora*) to 617 bp (*P*. *vulgaris* s.l.). The sister group taxa had relatively variable sequences in length, with ITS sequences ranging from 501 bp (*Utricularia intermedia*) to 650 bp (*Genlisea violacea*).

After introducing the necessary gaps, the ITS_T_ alignment resulted in a matrix of 695 characters, of which 200 constant and 495 variable (425 potentially parsimony-informative). The mean G+C content of the ITS matrix was 63.5%. Among the *Pinguicula* species sequenced in this study, the lowest G+C content was found in some *P*. *hirtiflora* accessions (P23, P24) (55.4%) and the highest in *P*. *balcanica* (P5, P6) (66%) (Table 1).

### Phylogenetic reconstructions

**Fig 2:**
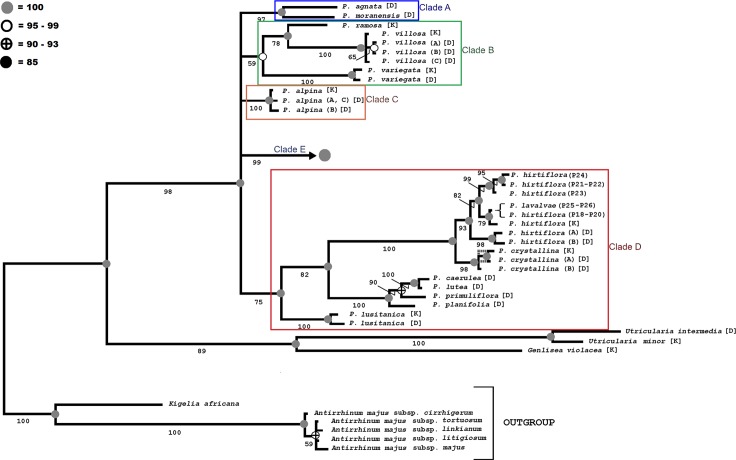
Bayesian phylogenetic tree of *Pinguicula* (clades A-D), reconstructed using ITS_T_ sequences. **Circles on the nodes show posterior probabilities (PP) from the Bayesian analysis under the GTR+I+G model (15,000,000 generations, burn-in 12%).** Grey circles: PP = 100; white circles: PP = 99–95; black/white circles: PP = 90–93; black circle: PP = 85. Branches present in the MP strict consensus tree is marked with a dashed line (MP trees = 2720, steps = 1605, CI = 0.57, RI = 0.89). Bootstrap percentages (1000 replicates) are given below the branches. D or K letters after the taxon refer to the accessions obtained from Degtjareva et al. [14] or Kondo & Shimai [15], respectively (Table 2). See Table 1 for further details about our *Pinguicula* accessions.

**Fig 3:**
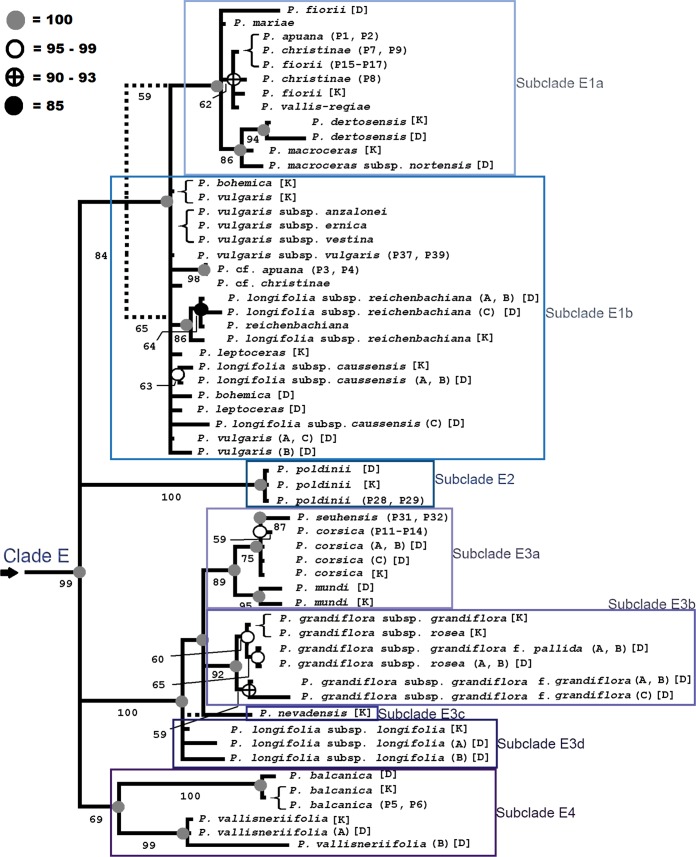
Bayesian phylogenetic tree of *Pinguicula* (clade E), reconstructed using ITS_T_ sequences. **Circles on the nodes show posterior probabilities (PP) from the Bayesian analysis under the GTR+I+G model (15,000,000 generations, burn-in 12%).** Grey circles: PP = 100; white circles: PP = 99–95; black/white circles: PP = 90–93; black circle: PP = 85. Branches present in the MP strict consensus tree is marked with a dashed line (MP trees = 2720, steps = 1605, CI = 0.57, RI = 0.89). Bootstrap percentages (1000 replicates) are given below the branches. D or K letters after the taxon refer to the accessions obtained from Degtjareva et al. [14] or Kondo & Shimai [15], respectively (Table 2). See Table 1 for further details about our *Pinguicula* accessions.

Inferred phylogenies from the BI and MP analyses of the ITS_T_ datasets produced a very similar topology with similar statistical support (Figs 2 and 3). The MP analysis yielded 492 most-parsimonious trees with a consistency index (CI) of 0.57 and retention index (RI) of 0.89. The strict consensus tree was 1592 steps long. The bootstrap support showed high values (BS > 70%) for the 72% of the nodes (Figs 2 and 3). The Bayesian tree with posterior probabilities (PP) is also shown in Figs 2 and 3. An unresolved basal structure for some *Pinguicula* groups is detected, albeit many clades are well supported (Figs 2 and 3).

A basal polytomy defined by five clades can be detected. The basally collapsed branches consist of three smaller groups (Clade A = *P*. *agnata*+*P*. *moranensis*; Clade B = *P*. *ramosa* Miyoshi+*P*. *villosa* L.+*P*. *variegata* Turcz. and Clade C = lineage of *P*. *alpina*) and a well-supported lineage (Clade D) where *P*. *hirtiflora/P*. *lavalvae/P*.*crystallina* are sister to *Pinguicula* species from the south-east United States of America (*P*. *caerulea*+*P*. *lutea* Walter+*P*. *primuliflora* C.E.Wood & R.K.Godfrey+*P*. *planifolia* Chapm.) and the European western-Atlantic *P*. *lusitanica*. In the next node (Clade E), which is not as well supported as other clades, four subclades can be identified. The first subclade (E1) includes *P*. *vulgaris* and allied taxa, with internal further subdivisions, including many Italian endemics (e.g. *P*. *apuana*, *P*. *fiorii*, and *P*. *christinae*), and other taxa related to *P*. *vulgaris*. The second subclade (E2) includes a single lineage with the Italian endemic *P*. *poldinii*. The third subclade (E3) includes further subdivisions of species with mostly Iberian origin, that will be discussed in more detail. Finally, the fourth subclade (E4) includes *P*. *balcanica* and *P*. *vallisneriifolia* Webb.

## Discussion

### Comparisons to previous phylogenetic reconstructions

Despite we could not include all taxa comprised in the genus *Pinguicula*, our sample size and array of species was sufficient to make phylogenetic assessments on the Italian endemic taxa (Figs 2 and 3). Comparing the phylogenies obtained with the ITS_T_ data published by Degtjareva *et al*. [14] and by Kondo & Shimai [15] and the *mat*K-*trn*K sequences published by Cieslak et al. [8], the overall topology of *Pinguicula* species is generally consistent, although showing several differences.

Cieslak *et al*. [8] in their *mat*K-*trn*K analysis, identified five main clades, partly overlapping with our results. Among the main similarities, the deep branching position of species belonging to our clade D (*P*. *lusitanica* and *P*. *crystallina*) and the overall placement of taxa allied to *P*. *vulgaris* in a single clade (corresponding to our clade E, Fig 3). Among the incongruences, *P*. *mundi* and *P*. *corsica* are placed on different lineages, whereas in our and in the previous ITS_T_ studies [14, 15] the aforementioned species are always placed in the same clade. *Pinguicula primuliflora* and *P*. *lutea* are included in the same clade of *P*. *lusitanica*, whereas in our analysis they form a distinct clade (B). The position of *P*. *alpina* is also in contrast with our results (clade C, Fig 2), since Cieslak *et al*. [8] place it in an isolated position within East Asian species (our deep branching clade D). The differences between our results and those published by Cieslak *et al*. [8] can be explained by the use of different molecular markers, as already mentioned in the Introduction.

No major differences are detected in the tree topologies with respect to previously published ITS_T_ studies. Kondo & Shimai [15] detected *P*. *crystallina* and *P*. *hirtiflora* as sister to all the remaining *Pinguicula* species. On the contrary, we found *P*. *lusitanica*, a species occurring on western coast of the Atlantic Ocean, from northern Morocco to Scotland [52], as belonging to the same, earliest branching, clade (D) (Fig 2). The rest of the phylogenetic reconstruction of Kondo & Shimai [15] is largely consistent with our finding, but included only two Italian endemics (*P*. *fiorii* and *P*. *poldinii*).

Generally, the topologies found by Degtjareva *et al*. [14] are also consistent with our findings. Similarly to Kondo & Shimai [15] and Cieslak *et al*. [8], they identified *P*. *crystallina* and *P*. *hirtiflora* as sister group to all other *Pinguicula* species. *Pinguicula lusitanica*, once again, is placed outside this clade. *Pinguicula alpina* is found in the same clade as *P*. *moranensis*, but these species belong to different clades in our results (A and C, respectively) (Fig 2).

### The taxonomical evaluation of *P*. *lavalvae*

All the species within clade D (Fig 2) show a tropical growth type [3], and have been classified within *P*. sect. *Cardiophyllum* [5]. Among them, *P*. *crystallina* occurs east of the Aegean sea in southern Turkey and Cyprus, while *P*. *hirtiflora* occurs west of the Aegean sea in the Balkans and in separate populations in southern Italy, mainly in Campania and a single population in Calabria [53, 54, 55, 56]. A large degree of variability and complexity is known in this group, with several infraspecific taxa described during the years for *P*. *hirtiflora* [5], and a recently described narrow-endemic to Turkey related to *P*. *crystallina*, i.e. *P*. *habilii* Yıldırım, Şenol & Pirhan [57], not included in our analysis. Given their unique basic chromosome number (*x* = 14) [5], it has been hypothesised that *P*. *crystallina/hirtiflora* may be the result of an ancient hybrid event involving a species similar to *P*. *lusitanica* (*x* = 6) and a species similar to *P*. *corsica* (*x* = 8). This hypothesis seems partly supported by our investigation, given the relative closeness of the *P*. *crystallina/hirtiflora* complex to *P*. *lusitanica*. Our data also confirm that *P*. *crystallina* and *P*. *hirtiflora* are separate species [7, 14].

It is interesting to note that there is a remarkable ITS_T_ molecular variation within *P*. *hirtiflora*. In particular, the accessions from Calabria (southern Italy) (P21-P22, Fig 2 and Table 1) fall in a clade distinct from *P*. *hirtiflora* (and *P*. *lavalvae*) from Campania (P18-P20 and P25-P26, respectively, Fig 2 and Table 1), as does also the alien population from south-eastern France, Val Roya (P24, Fig 2 and Table 1) [54]. This likely attests (a) for a Balkan origin of the population from Calabria, (b) for a Balkan (or Calabrian) artificial origin for the alien population occurring in south-eastern France, known since the 2000s [54]. It can be speculated that the newly found alien populations of *P*. *hirtiflora* in the Czech Republic [58] and Switzerland [56] also derive from commercialized Balkan populations.

*Pinguicula lavalvae* was recently described as a narrow endemic to Sabato Valley, Mts. Picentini (Campania, southern Italy) [23]. The authors compared it with *P*. *hirtiflora* from different portions of its range, distinguishing *P*. *lavalvae* for several phenotypic features of the corolla and calyx. Despite this, the latter species showed a complete sequence identity with the accessions of topotypical *P*. *hirtiflora* from Campania [59]. The ITS sequence, along with the geographic vicinity of the two taxa, points to an assignment of *P*. *lavalvae* to an intraspecific rank within *P*. *hirtiflora*. Indeed, Fleischmann [56] considered *P*. *lavalvae* as simply one of the many white-flowered variants (*P*. *hirtiflora* f. *pallida*) of *P*. *hirtiflora*, as those that are found in the Balkans and elsewhere in Italy [60]. Nevertheless, the ITS_T_ variation in all accessions from Campania compared to the Balkans and an ecological niche shift of these populations (M. Innangi *et al*. in preparation) highlights the necessity of more investigation before establishing taxonomical ranks.

### Subclade E1a: the clade comprising most Apennine endemics

A single clade unites four of the *Pinguicula* sect. *Pinguicula* species endemic to the Apennines: *P*. *apuana*, *P*. *christinae*, *P*. *fiorii*, and *P*. *vallis-regiae*. Only one GenBank accession of *P*. *fiorii* is falling outside this clade (Fig 3), but it must be noted that this accession was obtained from seeds [14], and may well represent a misidentification or confusion of materials. This clade includes also another Italian endemic species, the tetraploid *P*. *mariae*. On a slightly different branch within the subclade, there are two other octoploid species, *P*. *dertosensis* (Cañig.) Mateo & M.B.Crespo, endemic to south-eastern Spain [61, 62] and *P*. *macroceras* Pall. Ex Link, endemic to northern America and Japan [7]. The four species endemic to Apennines have very similar sequences, which are identical in some case (i.e. *P*. *apuana*, *P*. *christinae* P7 and P9, and *P*. *fiorii* P15-P17), clearly pointing out to a common origin. However, given the clear qualitative and quantitative combination of character-states that has been used to distinguish these *taxa*, coupled with allopatry [20, 21, 22], we deem reasonable to maintain them at species level. As a matter of fact, the discriminating resolution of ITS may not be enough within such level of variability. Thus, further intrapopulation approaches could be necessary. The collocation in this clade of *P*. *vallis-regiae*, morphologically very similar to *P*. *poldinii* [20], was quite unexpected. Indeed, the latter species represents a well distinct lineage in the tree (see further in the text).

Both *P*. *christinae* and *P*. *fiorii*, show a certain morphological affinity with *P*. *balcanica* (endemic to Balkan Peninsula) (see [22, 18], respectively). According to our results, such relationships can be excluded, given the position of *P*. *balcanica* in a completely different clade (E4, Fig 3), as sister to the heterophyllous (i.e. bearing spring and summer rosettes significantly differing in shape and dimensions) *P*. *vallisneriifolia*, a peculiar Spanish endemic [61, 62]. Finally, *P*. *apuana* was compared by Ansaldi & Casper [21] with *P*. *leptoceras* Rchb., endemic to Alps [7] and with the circumboreal *P*. *vulgaris* [7]. The relationship with these species can also be excluded, given that both *P*. *leptoceras* and *P*. *vulgaris* fall in a different clade (E1b, Fig 3).

### Subclade E1b: the clade of P. vulgaris and its allies

Albeit this clade is weakly supported and highly polytomic (Fig 3), several phylogenetic inferences can be provided. *Pinguicula vulgaris*, a widespread Circumboreal octoploid species, forms a clade together with similar taxa such as *P*. *bohemica* Krajina, a tetraploid/octoploid endemic to the Czech Republic [5, 63], the heterophyllous tetraploid *P*. *reichenbachiana* (endemic to the Maritime Alps), *P*. *longifolia* subsp. *caussensis* Casper (endemic to southern France) and the tetraploid Alpine endemic *P*. *leptoceras* [7], provisionally including the plants recognised as “*P*. *arvetii*” in SW Alps [64]. According to Conti & Peruzzi [20], *P*. *vulgaris* segregated into geographical races at its range borders. In the case of three of them, in central Italy, taxonomic recognition at subspecies level was proposed: *P*. *vulgaris* subsp. *anzalonei*, *P*. *vulgaris* subsp. *ernica* and *P*. *vulgaris* subsp. *vestina*. It is interesting to note that the three taxa share identical sequences, but collectively differ from other *P*. *vulgaris* accessions for two SNP’s (Single Nucleotide Polymorphism). This likely attests for their common origin, but given their clear diagnosability and allopatry, also in this case we deem the original subspecific rank as appropriate.

Concerning the accession of *P*. cf. *apuana* from Bendola (SE France), our results clearly highlight for a significant molecular differentiation of this population, within the clade including *P*. *vulgaris* and its allies. The taxonomic position of this population needs further investigation. As concerns *P*. cf. *christinae* from Val D’Aveto (Liguria), also falling in the same clade as *P*. *vulgaris* accessions, morphological evidence was gathered to support its difference from both *P*. *christinae* and *P*. *vulgaris*. For further detail, see floral morphometric analysis in S1 File. The *Pinguicula* population occurring in Val d’Aveto (Liguria), at first sight, shows morphological affinity with *P*. *christinae*, but also some character-states intermediate or similar to those of *P*. *vulgaris* s.l. Our molecular results highlight a phylogenetic affinity with the latter species, but also a molecular differentiation of this population in the ITS_T_ sequences (three SNP’s). In addition, our morphometric study of floral features clearly attests for a distinctive combination of character-states in these plants (S1 File). Accordingly, and given the allopatry of this population from both *P*. *vulgaris* s.l. and *P*. *christinae*, we describe here this population as a new narrow endemic species:

***Pinguicula lattanziae*** Peruzzi & Gestri ***sp*. *nov*.** (urn:lsid:ipni.org:names:XXXXXXXX-X)

Type: Liguria, N Apennine: Monte Aiona, 1600–1700 m a.s.l., 21 June 2014, *G*. *Gestri et C*. *Gavazzi s*.*n*. (holotype, PI).

The new taxon is close to *P*. *christinae* and *P*. *vulgaris*, but distinct for its narrower corolla upper lobes (2.5 ± 0.76 mm, not 4.72 ± 0.81 mm and 3.44 ± 0.69 mm, respectively) and narrower corolla median lobe (2.08 ± 0.42 mm, not 3.31 ± 0.67 mm and 3.83 ± 0.65 mm, respectively). It shares with *P*. *vulgaris* the corolla opening angle (≤ 90°) and the spur length (5–7 mm). This new species is dedicated to Edda Lattanzi (Rome), expert in Italian flora, in occasion of her 85^th^ birthday.

### Subclade E2: the status of *Pinguicula* ser. *Prealpicae*

Casper in Ansaldi & Casper [21] formally described *Pinguicula* ser. *Prealpicae*, to accommodate *P*. *poldinii* (type species), *P*. *mariae* and–putatively–*P*. *vallis-regiae*. The shared character-states used to justify this proposal were: homophyllous rosettes (i.e. spring and summer rosettes not significantly differing in shape and dimensions), widely open corollas with long spur and tetraploid status. The same authors explicitly exclude *P*. *reichenbachiana* from this series for being heterophyllous, regardless of other morphological similarities. According to our phylogenetic results, *P*. *mariae* and *P*. *vallis-regiae* represent two distinct lineages, placed in a large clade where also *P*. *apuana*, *P*. *christinae*, and *P*. *fiorii* occur (subclade E1a, Fig 3). On the contrary, the type species *P*. *poldinii* lies in a very distinct, albeit unresolved, lineage (subclade E2, Fig 3), to which eventually should be restricted the application of the ser. *Prealpicae*. However, this is not justified at all by morphology, and this highlights that certain characters in *Pinguicula* (i.e. homophyllous vs. heterophyllous rosettes, chromosome number) are useful taxonomic markers at species level, but cannot be safely used *per se* to establish systematic/evolutionary relationships.

### Subclade E3: the phylogenetic placement of *P*. *sehuensis* and allied species, with some remarks on *P*. *longifolia* s.l.

The discovery of a new *Pinguicula* in Sardinia was defined as “*the most important finding of a butterwort during the past 50 years in Europe*” (J.S. Casper, in litt.). *Pinguicula sehunensis* was recently described by Bacchetta *et al*. [1] as morphologically close to *P*. *dertosensis*, *P*. *nevadensis* (H.Lindb.) Casper–both endemic to Spain [61, 62]–and the western European *P*. *grandiflora* Lam. [7]. According to our results, *P*. *sehuensis*, endemic to Sardinia, shares instead a common origin with *P*. *corsica*, endemic to Corsica (Fig 3). This phylogenetic structure parallels the biogeographic closeness between the two species, which also share a diploid status. Whereas *P*. *dertosensis* is placed in a completely different subclade (E2a, Fig 3), *P*. *nevadensis* and *P*. *grandiflora* belong to the same clade including also *P*. *corsica*+*P*. *sehuensis*, together with *P*. *mundi* Blanca, Jamilena, Ruiz Rejón & Reg.Zamora, also endemic to south-eastern Spain [61, 62]. The position of *P*. *mundi* as sister species to *P*. *corsica* and *P*. *sehuensis* is biogeographically significant, as until 25 Ma, in the late Oligocene, Sardinia and Corsica started to drift from modern Provence and reached their current geographical position in the middle Miocene (ca. 15 Ma) [64]. The ploidy level and ITS sequences of both *P*. *corsica* and *P*. *sehuensis* suggest that both species derived from a common diploid ancestor in the geologically and ecologically stable environments of the Hercynian massif of Corsica and Sardinia sometime between 25 and 15 Ma. Accordingly, and following the criteria proposed by Siljak-Yakovlev & Peruzzi [65], it is possible to hypothesize for *P*. *corsica* and *P*. *sehuensis* the status of patro-schizoendemic taxa.

*Pinguicula grandiflora* s.l., *P*. *nevadensis* and *P*. *longifolia* subsp. *longifolia* fall within the same subclade, and they are tetraploid. As argued for *P*. *grandiflora*, a species with Lusitanian distribution, its ploidy level and current distribution has been shaped by palaeoclimatic Quaternary events [66]. Our results further suggest that Quaternary glaciations played a prominent role in the evolution of polyploid butterworts currently scattered and isolated in the mountains.

Finally, some remarks can be done for *P*. *longifolia* s.l. The three different subspecies of *P*. *longifolia* are clearly paraphyletic, as already demonstrated by Cieslak *et al*. [8]. While *P*. *longifolia* subsp. *reichenbachiana*, endemic to few locations on the Maritime Alps in France and Italy, was already considered as a distinct species by several authors [1, 20, 21, 22], this is not the case for *P*. *longifolia* subsp. *caussensis*. The latter taxon, endemic to Les Causses in southern France, is clearly not related to *P*. *longifolia* s.str. and it has several quali-quantitative features that distinguish it as much as it happens for *P*. *reichenbachiana* [67, 68]. Degtjareva *et al*. [14] already pointed out the necessity of redefining the taxonomic state of *P*. *longifolia* subspecies. Thus, we deem appropriate to change its taxonomic status to species level as ***P*. *caussensis*** (Casper) Innangi, De Castro & Peruzzi ***stat*. *nov*.** (Bas.: *P*. *longifolia* subsp. *caussensis* Casper, Biblioth. Bot. 31(127–128): 154. 1966).

## Conclusions

We can summarize our results as follows:

1. We identified a basal polytomy defined by five clades in *Pinguicula*. All of the Italian endemic taxa (with the exception of *P*. *lavalvae*) fall within the large clade E, which corresponds to *P*. sect. *Pinguicula* (type species: *P*. *vulgaris*).
2. Among Italian endemics, *P*. *poldinii* represents the most distinct lineage. Other species, such as *P*. *apuana*, *P*. *christinae*, *P*. *fiorii*, *P*. *mariae*, and *P*. *vallis-regiae* show strong molecular similarities and form a single subclade, although their taxonomic rank can be retained. The subspecies of *P*. *vulgaris* endemic to Italy (i.e. *P*. *vulgaris* subsp. *anzalonei*, *P*. *vulgaris* subsp. *ernica*, and *P*. *vulgaris* subsp. *vestina*) all share similar sequences, but are sufficiently different from subsp. *vulgaris* and are allopatric, thus can be considered as valid taxa. *Pinguicula sehuensis* is closely related to *P*. *corsica*, but it is clearly a good taxon that highlights interesting biogeographic patterns. *Pinguicula lavalvae* shares sequences with *P*. *hirtiflora* from Campania and could be potentially considered a subspecies of the latter, but the whole group of *P*. *hirtiflora* needs further investigation before a final taxonomic setting can be proposed. Finally, this study contributed to the identification of a new narrow Italian endemic systematic unit in Clade E, i.e. *P*. *lattanziae* sp. nov., a still undescribed species from SE France, and the change in taxonomical status from *P*. *longifolia* subsp. *caussensis* to *P*. *caussensis*.

In conclusion, our research allowed broadening the knowledge of taxonomic and evolutionary trends in the whole genus *Pinguicula*, with a special focus on the Italian endemics. The genus *Pinguicula* is complex and evolutionarily interesting, thus further research, possibly uniting different disciplines (e.g. population genetics, morphometrics, ecology and karyology), is still needed to clarify the taxonomic value of several taxa and implement the knowledge of evolutionary and biogeographic patterns in the whole genus.

## Supporting Information

S1 File Floral morphometric analysis of *Pinguicula* cf. *christinae* from Val d’Aveto. (DOCX)
