# Supplementary material for: Disentangling Phylogenetic Relationships in a Hotspot of Diversity: The Butterworts (Pinguicula L., Lentibulariaceae) Endemic to Italy
Source: PLoS One. 2016 Dec 28;11(12):e0167610. doi: 10.1371/journal.pone.0167610 (PMC5193322; doi:10.1371/journal.pone.0167610)
Supplement: S1 File — (DOCX) [file pone.0167610.s001.docx]

**S1 File.**

**Floral morphometric analysis of *Pinguicula* cf. *christinae* from Val d’Aveto.**

Floral morphometry was studied by measuring in the field ten quantitative continuous floral characters on 19 individuals from the studied population (P10, see Table 1): a) corolla length; b) spur length; c) upper lobes length; d) upper lobes width; e) lower lip central lobe length; f) lower lip central lobe width; g) lower lip lateral lobes length; h) lower lip lateral lobes width; i) calyx upper lip length; j) calyx lower lip length. Floral morphometric data about the population from Val d’Aveto were compared with those of other apparently morphologically close taxa (*P. christinae* and *P. vulgaris* s.l.). The data about the latter two species were derived from Peruzzi and Gestri [1].

After logarithmic transformation, these quantitative data were subjected to Discriminant Analysis, by means of PAST ver. 3.03 software [2, 3], with the individuals a priori assigned to the three taxa based on morphological features and geographical distribution. Each character was also subjected to univariate analysis (ANOVA or Kruskal-Wallis test with corrections for multiple comparisons, Tukey HSD test and Bonferroni, respectively), by means of the same software.

Discriminant Analysis resulted in 97.59% (jackknifed) correct classification of individuals a priori attributed to *P. christinae*, *P. vulgaris* s.l. and the population from Val D’Aveto (Fig. 1). There is possible confusion between *P. christinae* and the plants from Val D’Aveto (2/83 individuals not correctly classified), between *P. vulgaris* s.l. and the plants from Val D’Aveto (1/19 individuals not correctly classified) and, less importantly, between *P. christinae* and *P. vulgaris* s.l. (only 1/64 individuals not correctly classified). This picture is confirmed by univariate analysis (Kruskal-Wallis with Bonferroni correction): characters #1, 2, 3, 5, 6, 7, 8, 10 are significantly different between the three groups (p < 0.01), the length of corolla median lobe (character #9) is not significantly different among the three groups, while the spur length (character #4) is shared between *P.* cf. *christinae* and *P. vulgaris* s.l.

**References**

1. Peruzzi L, Gestri G. A new butterwort species (*Pinguicula*, Lentibulariaceae) from Northern Apennine (Italy). Pl Biosyst. 2013; 147: 692-703.

2. Hammer Ø, Harper DAT, Ryan PD PAST: Paleontological Statistics sofware package for education and data analysis. Paleontologia Electronica*.* 2001; 4: 1-9.

3. Hammer Ø. PAST 3.03. 2014. Available at http://folk.uio.no/ohammer/past. Accessed 10 October 2014.


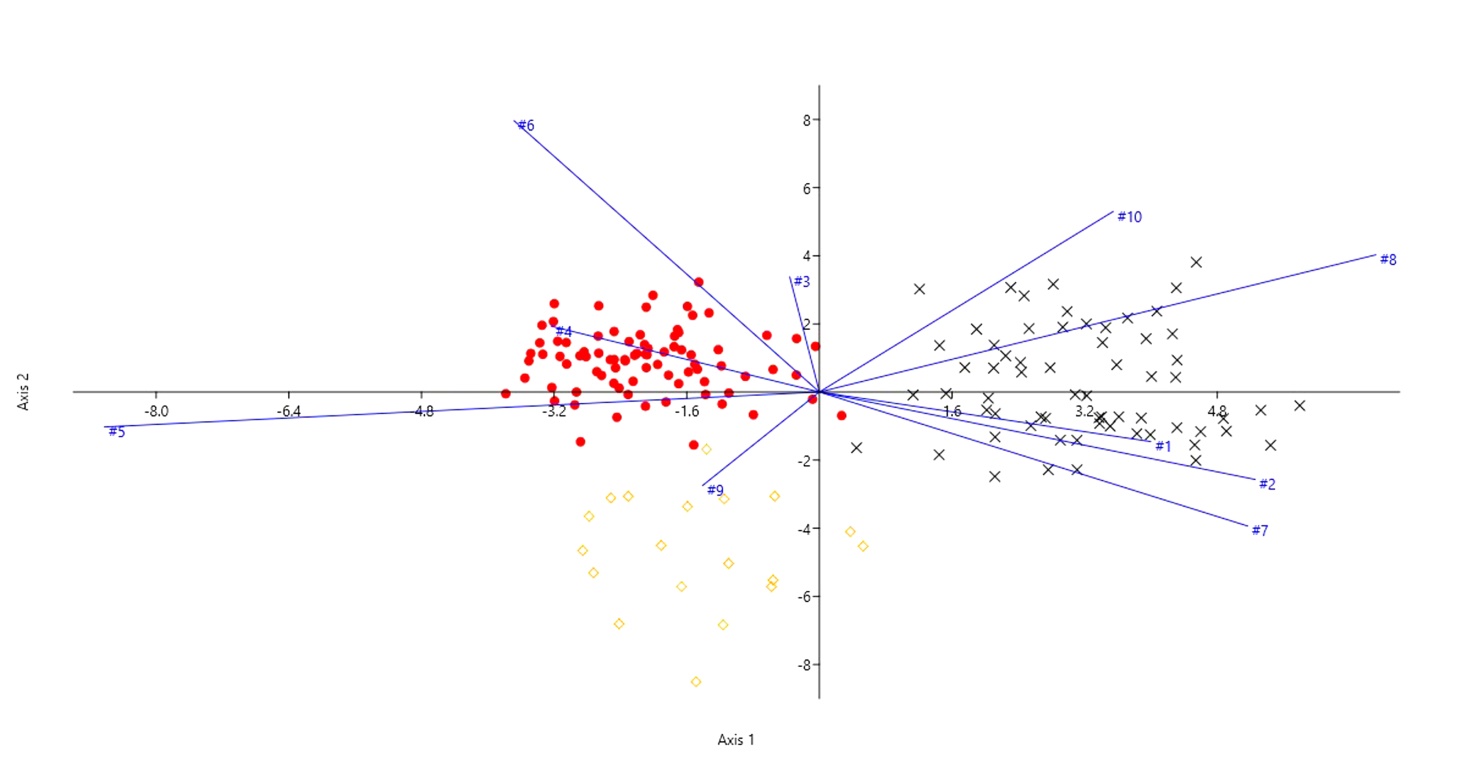


**Fig. 1.** **Disciminant Analysis biplot based on the 10 considered flower morphological characters, with 3 *a priori* identified groups corresponding to *Pinguicula christinae* (filled red dots), *P. vulgaris* s.l. (black crosses), *P.* cf. *christinae* from Val d’Aveto (empty orange squares).** The character numbers refer to the list in Material and Methods: a) corolla length; b) spur length; c) upper lobes length; d) upper lobes width; e) lower lip central lobe length; f) lower lip central lobe width; g) lower lip lateral lobes length; h) lower lip lateral lobes width; i) calyx upper lip length; j) calyx lower lip length.
